# Temporal changes in access to FRAX® in Thailand between 2010 and 2018

**DOI:** 10.1007/s11657-019-0613-2

**Published:** 2019-06-21

**Authors:** Pojchong Chotiyarnwong, Nicholas C Harvey, Helena Johansson, Enwu Liu, Mattias Lorentzen, John A Kanis, Eugene V McCloskey

**Affiliations:** 1 Department of Orthopaedic Surgery, Faculty of Medicine, Siriraj Hospital, Mahidol University, Bangkok, 10700 Thailand; 2 Academic Unit of Bone Metabolism, Department of Oncology and Metabolism, The Mellanby Centre For Bone Research, University of Sheffield, Sheffield, UK; 3 MRC Lifecourse Epidemiology Unit, University of Southampton, Southampton, UK; 4 NIHR Southampton Biomedical Research Centre, University of Southampton and University Hospital Southampton NHS Foundation Trust, Southampton, UK; 5 Centre for Metabolic Diseases, University of Sheffield Medical School, Beech Hill Road, Sheffield, S10 2RX UK; 6 Centre for Bone and Arthritis Research (CBAR), Sahlgrenska Academy, University of Gothenburg, Gothenburg, Sweden; 7 Mary MacKillop Institute for Health Research, Australian Catholic University, Melbourne, Victoria Australia; 8 Region Västra Götaland, Geriatric Medicine Clinic, Sahlgrenska University Hospital, Gothenburg, Sweden; 9 Geriatric Medicine, Department of Internal Medicine and Clinical Nutrition, Institute of Medicine, Sahlgrenska Academy, University of Gothenburg, Gothenburg, Sweden; 10 Centre for Integrated Research into Musculoskeletal Ageing, University of Sheffield Medical School, Sheffield, UK

**Keywords:** FRAX, Usage, Session, Thailand, Google Analytics

## Abstract

**Summary:**

The usage of FRAX® tool in Thailand and other countries was explored using Google Analytics data. Over the period 2010–2018, Thailand ranked 35th in the world for FRAX usage (the US is ranked first). Incorporation of FRAX into a national osteoporosis guideline in Thailand appears to have increased its usage.

**Purpose:**

To document access to the web-based FRAX® tool and specifically its access in Thailand between 2010 and 2018.

**Methods:**

A descriptive retrospective study using data from Google Analytics that provides numerical and geographical information on internet access to the FRAX tool website worldwide.

**Result:**

In Thailand, Bangkok is the highest ranked site for FRAX access with more than 20,000 usage sessions since 2010 (3.6 usage session per 1000 population) followed by Khon Kaen and Chiang Mai. It has been accessed from within 76 out of 77 provinces (98.7%). There was a steady increase in access to FRAX from within Thailand of approximately 1000 usage sessions per year between 2010 and 2016. After the FRAX fracture risk calculation was included in the national guideline for osteoporosis management published in late 2016, the rate of increase in access was four-fold higher compared with the previous period. In world ranking, the USA is the country with the most frequent access to the FRAX tool, whereas Thailand was ranked 35th in the world. There were weak but significant correlations between the absolute number of FRAX sessions and population size (*r* = 0.165, *p* = 0.011) and land area (*r* = 0.375, *p* < 0.001).

**Conclusion:**

Access to the FRAX tool website is increasing in Thailand. The incorporation of FRAX into national guidelines, in parallel to the adoption of osteoporosis fracture prevention into national policy, has had a rapid and significant impact on its use.

**Electronic supplementary material:**

The online version of this article (10.1007/s11657-019-0613-2) contains supplementary material, which is available to authorized users.

## Introduction

Osteoporosis is one of the most common health problems in older people, and its consequence, fragility fractures, can have large impacts on the patient and their family [1]. There is an increasing recognition of the need to intervene in patients at high risk of fracture to decrease this fracture burden. The FRAX® tool, developed by researchers at the University of Sheffield, is used to evaluate individual fracture risk, based on models that integrate clinical risk factors with or without additional information provided by bone mineral density (BMD) at the femoral neck [2]. It is freely and easily accessible to healthcare workers with internet access (www.sheffield.ac.uk/FRAX) and is also available as a paid for application on smartphones. Individual data input and analysis takes less than 1 min. FRAX calculates the 10-year probability of hip fracture alone and of a major osteoporotic fracture (clinical spine, forearm, hip, or shoulder fracture) in patients between 40 and 90 years of age. First released in 2008, FRAX currently has models calibrated for 63 countries, comprising 69 populations (China, Singapore, and the USA have several ethnic-specific models) and is available in 34 languages (Chinese and Portuguese have 2 difference versions).

Whereas FRAX generates numerical fracture probabilities, guidance is needed over the interpretation of these numbers so that its clinical utility is driven by its incorporation into clinical guidelines. To date, FRAX has been accepted in many osteoporosis guidelines in many regions of the world [3–7]. Given disparities in fracture incidence, health policy, and budgetary priorities, there is no international consensus on FRAX thresholds either for the use of BMD or an intervention threshold to be used as an indicator for treatment. In Thailand, FRAX was first mentioned in a guideline launched in 2010 [8], wherein a FRAX calculation was recommended when the femoral neck BMD T-score was between − 1.0 and − 2.5, and osteoporosis treatment initiated if the 10-year probability of fracture at hip was ≥ 3% or major osteoporosis fracture ≥ 20%, mirroring recommendations from the USA [9]. In 2010, in the absence of a Thailand-specific FRAX model, it was recommended to use the US Asian or Japanese models. Subsequently, a model for Thailand was released in May 2013, but a new guideline was not published until the end of 2016 [6]. In this latest guideline, there are several indications for the initiation of pharmacological treatment of osteoporosis. In general, for postmenopausal osteoporosis and male idiopathic osteoporosis age over 50 years, the indication can be one of the following: presentation with a hip or vertebral fracture from low-energy injury; lumbar spine BMD or femoral neck BMD or total hip BMD showing T-score ≤ − 2.5; 10-year probability of hip fracture by FRAX using Thai model (with BMD or without) ≥ 3%.

The primary objective of this study was to assess the access of the online FRAX tool from Thailand and other countries across the period from 2010 to the end of 2018. A secondary objective was to determine, if possible, the impact of guideline incorporation in Thailand on FRAX usage.

## Methods

This is a retrospective descriptive study using a free tool, Google Analytics (https://analytics.google.com/analytics/web/) [10, 11], to document access of users worldwide to the FRAX calculator website. The survey of usage was conducted between 15 February 2010 (the earliest date available in this version of Google Analytics) and 31 December 2018. Data tables were exported from Google Analytics in XLSX format for further analysis. In Analytics, a session is the period of time a user is actively engaged with FRAX tool website. All usage data are associated with a session, whereas a bounce rate captures the percentage of visits in which a person leaves a FRAX website from the landing page without browsing any further, i.e., enter and then leave the FRAX website without a fracture risk calculation. The number of sessions per 1000 population was calculated from the total number of sessions divided by the population size and multiplied by 1000. An estimate of actual FRAX “usage sessions” was calculated from the total session multiplied by 1-bounce rate. The most recent Thai population data in 2017 were acquired from the Bureau of Registration Administration of Thailand [12]. FRAX usage was assessed in Thailand and compared with patterns of use elsewhere in the world. The land area and approximate 2018 population for each country were acquired from the latest United Nations Population Division estimates, with permission [13]. All graphs and table were made using the Microsoft office 2016 package (Microsoft WA, USA). Pearson correlation statistics were analysed using IBM SPSS version 18.0 (SPSS Inc., Chicago, Illinois, USA).

## Results

### Usage of FRAX tool in Thailand

**Fig. 1:**
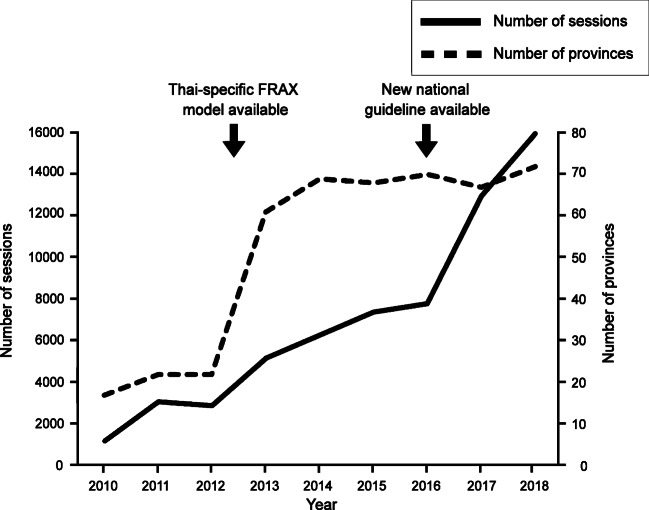
Graph showing the number of Thailand provinces (77 provinces in total) accessing FRAX and the number of total sessions of FRAX accessed from Thailand, recorded per year during the current analysis period (2010–2018)

**Fig. 2:**
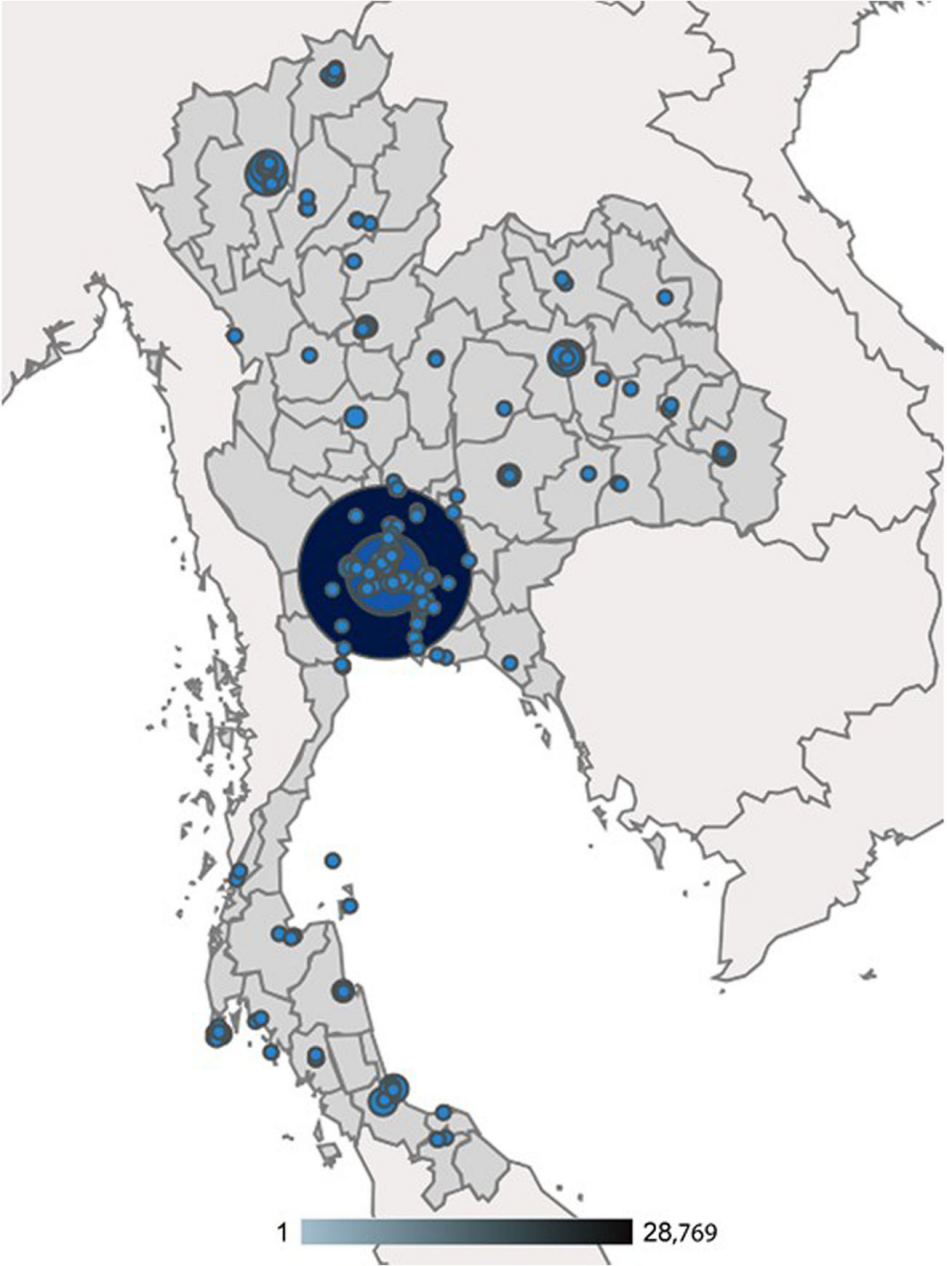
Google Analytics output of FRAX access and usage across cities in Thailand over the period of the study (2010–2018). The size of the circle reflects the number of usage episodes recorded

Thailand currently comprises 77 regions altogether (76 provinces + Bangkok metropolitan). In the time period studied here (15 February 2010–31 December 2018 inclusive), the first two sessions of access to FRAX from Thailand, recorded in this version of Google Analytics, were on 2 July 2010 from Bangkok and Khon Kaen. Within 2010, FRAX had been accessed from 17 of the Thailand regions and this increased rapidly to a plateau of 69–71 regions in 2014 (Fig. 1). The highest number of FRAX sessions arose from Bangkok, followed by Khon Kaen and Chiang Mai (Table 1, Fig. 2). The number of sessions rose steadily from 2010 to 2016, with an approximate seven-fold increase over this time, reflecting an average increase in session rate of 1098 per year. In 2017, there was an apparent acceleration in FRAX session access so that over the last two years there has been an average increase of 4079 sessions per year. In terms of sessions recorded on the FRAX website during the period of this study, Thailand is ranked 35 out of 239 countries or dependencies (Table 2). A fully detailed account of FRAX access from Thailand since 2010 is provided in Supplementary Table 1.

**Table 1:** The top ten Thailand provinces accessing the FRAX tool between February 2010 and December 2018, rank by FRAX usage session

Rank	Region (province)	Sessions	2017 population	Session per 1000 population	Bounce rate	FRAX usage sessions	FRAX usage session per 1000 population
1	Bangkok	41,383	5,682,415	7.3	50.4%	20,521	3.6
2	Khon Kaen	3694	1,805,910	2.0	35.2%	2394	1.3
3	Chiang Mai	3582	1,746,840	2.1	49.6%	1806	1.0
4	Chon Buri	2110	1,509,125	1.4	52.0%	1013	0.7
5	Songkhla	1322	1,424,230	0.9	52.5%	628	0.4
6	Nonthaburi	834	1,229,735	0.7	45.9%	451	0.4
7	Phitsanulok	745	865,368	0.9	46.9%	396	0.5
8	Nakhon Ratchasima	766	2,639,226	0.3	52.4%	365	0.1
9	Nakhon Pathom	638	911,492	0.7	47.3%	336	0.4
10	Samut Prakan	688	1,310,766	0.5	53.5%	320	0.2

**Table 2:** The top ten countries plus Thailand accessing the FRAX tool between February 2010 and December 2018, rank by FRAX usage session

Rank	Country	Sessions	2018 population	Sessions per 1000 population	Bounce rate	FRAX usage sessions	FRAX usage sessions per 1000 population
1	USA	8,048,970	326,766,748	24.6	47.1%	4,258,344	13.0
2	UK	2,119,471	66,573,504	31.8	37.8%	1,317,755	19.8
3	Canada	646,607	36,953,765	17.5	48.5%	332,875	9.0
4	Spain	476,640	46,397,452	10.3	43.8%	268,052	5.8
5	Slovenia	269,829	2,081,260	129.6	13.8%	232,647	111.8
6	Japan	476,069	127,185,332	3.7	56.9%	205,401	1.6
7	France	312,778	65,233,271	4.8	41.6%	182,794	2.8
8	Sweden	265,260	9,982,709	26.6	35.4%	171,250	17.2
9	Brazil	252,299	210,867,954	1.2	34.0%	166,491	0.8
10	Belgium	280,156	11,498,519	24.4	48.8%	143,426	12.5
35	Thailand	62,353	69,183,173	0.9	49.3%	31,617	0.5

### Worldwide usage of FRAX tool

Usage of FRAX elsewhere in the world was assessed to provide a context for activity in Thailand. Over the period of this study, 228 countries or dependencies worldwide had documented access and interacted with the FRAX tool website. As previously reported, the country with the most frequent access to the FRAX tool website is the USA [14]. Over the reporting period, around 4.3 million usage sessions were conducted from the USA, followed by the UK (1.3 million) and Canada (0.3 million) (Table 2). Overall, the correlation (*r*) between usage session number and land area was low (*r* = 0.374, *p* < 0.001) and that with population size lower still (*r* = 0.164, *p* = 0.011). When usage was adjusted for population size, Slovenia showed the highest usage of FRAX (112 usage sessions per 1000 population), followed by Bermuda (91 usage sessions per 1000 population) and Malta (70 usage sessions per 1000 population). Within the top ten countries for absolute numbers of usage sessions listed in Table 2, following Slovenia, the highest rates adjusted for the population were seen in the UK and Sweden. The lowest usage session rate was seen in Brazil (0.8 usage session per 1000 population) and an even lower rate was recorded from Thailand (0.5 usage per 1000 population). A fully detailed account of FRAX access from all countries since 2010 is provided in Supplementary Table 2.

## Discussion

Like the developed world in general, Thailand is moving rapidly towards an aged society. It is estimated that in 2021, 13 million of the total 65 million population (i.e., 20%) will comprise men and women aged 60 years and older; by 2031 [15], it is predicted that this will rise to 28% of the total population. The impact that this will have for the burden of fractures has meant that osteoporosis is now recognised in national health policy in Thailand in 2018, under a campaign of “refracture prevention” that promotes secondary fracture prevention but also embraces osteoporosis screening and treatment [16]. Given the low availability of DXA and its relatively high associated costs (approximately US$ 60 for both lumbar spine and hip scans), it is highly unlikely that a campaign with DXA as the first line tool in Thailand would be achievable. In the UK and other European guidance, clinical risk assessment by the FRAX tool is advocated as the first line, with DXA measured BMD targeted to those who lie close to an intervention threshold [3, 17]. Such an approach shows a more economic use of scanning resources [18]. The pattern of FRAX usage over time in Thailand is interesting. For the first 2–3 years of the study period, a Thailand-specific model of FRAX was not available and users were advised to undertake calculations using the Japanese or US Asian tool [8, 19]. It is notable that the number of regions accessing FRAX increased quite dramatically in 2013, coinciding with, but perhaps unrelated to, the launch of the Thailand FRAX model. As noted above, the more rapid increase in actual FRAX usage occurred in line with updated guidance and a greater focus on the burden of osteoporosis in 2016/2017 [6]. Importantly, this new osteoporosis management guideline advocated the use of FRAX as a potential gateway to treatment; even in the absence of a DXA BMD measurement, as in menopausal woman and men aged over 50 years old, therapy can be indicated by a 10-year FRAX Thailand model probability of hip fracture of 3.0% or greater. Of note, a recent study in the UK that used FRAX as a population screening tool, in combination with appropriate intervention in those at high risk of hip fracture, showed a 28% reduction in hip fracture [17, 20].

This retrospective descriptive study shows that the FRAX website–based tool is accessed worldwide. As expected, this access varies considerably from country to country; the finding that land area and, more importantly, population size are only relatively weakly correlated with usage suggests that other factors impact on FRAX website access. Awareness of osteoporosis, availability of resources, and the priority of fracture burden as a local or national healthcare concern are also likely to be of importance. For example, the availability of DXA equipment, if regarded as a surrogate measure of the awareness of osteoporosis and its priority, might be expected to relate to FRAX usage; Slovenia, the highest user based on population size, also has one of the highest concentrations of DXA scanners with 20 per million of the population compared with for example the UK (8.2 per million) [21]. In Thailand, where DXA provision is even more limited (0.67 scanners per million) [22], the FRAX usage is also much lower. It is of interest, however, that FRAX usage in Thailand is growing and the potential reasons for this are undoubtedly of interest; our observation of an approximately 4-fold increase in annual growth rates for FRAX access that followed the launch of new national guidelines in late 2016 suggest that national initiatives and specific guidance on FRAX’s role in risk assessment and management can enhance its usage.

Our study has some limitations. Firstly, the data from Google Analytics records access to the website, not the actual fracture risk calculation. Nonetheless, the session rate will still provide information on the pattern of use over time and across geographies. We have tried to remove accidental or transient site access by also analysing the sessions adjusted for the bounce rate; the patterns are similar though the absolute number of sessions is decreased. As the website counter embedded on the FRAX website actually documents full calculations, we have been able to estimate that there is an approximate ratio of 2.7 between actual calculations and usage (non-bounce) sessions. There is of course the possibility that the use of the online FRAX calculator may be for purposes other than patient evaluation and clinical care, for example, research or other academic purposes. However, it is likely that there is a correlation between clinical and research use within particular countries. A further point in the interpretation of high usage per capita is the impact of the overall population size; for example, the populations of Bermuda and Malta are relatively small, ranging from approximately 60,000–400,000, so that the adoption of FRAX by a relatively small number of clinicians can have a significant impact on the per capita use. Finally, the website is only one source of access to FRAX calculations; those conducted on densitometers or by a smartphone app or paper-based systems will not be included in our analysis.

## Conclusion

The FRAX online tool is widely accessed across the world. In Thailand, the use of FRAX has steadily increased over the period 2010–2018. A more rapid increase in the last 2 years suggests that its incorporation into national guidance in 2016 enhanced its usage. Further national initiatives to promote awareness should be strongly considered.

## Electronic supplementary material

ESM 1 (PDF 78 kb)

ESM 2 (PDF 152 kb)

## References

[1] Harvey N, Dennison E, Cooper C (2010). Osteoporosis: impact on health and economics. Nat Rev Rheumatol.

[2] Kanis JA, Oden A, Johansson H, Borgstrom F, Strom O, McCloskey E (2009). FRAX and its applications to clinical practice. Bone.

[3] Kanis JA, Harvey NC, Cooper C, Johansson H, Oden A, McCloskey EV, Advisory Board of the National Osteoporosis Guideline G (2016) A systematic review of intervention thresholds based on FRAX: a report prepared for the National Osteoporosis Guideline Group and the International Osteoporosis Foundation. Arch Osteoporos 11 (1):25. doi:10.1007/s11657-016-0278-z, 2016 10.1007/s11657-016-0278-z PMC4978487 27465509

[4] Buckley L, Guyatt G, Fink HA, Cannon M, Grossman J, Hansen KE, Humphrey MB, Lane NE, Magrey M, Miller M, Morrison L, Rao M, Robinson AB, Saha S, Wolver S, Bannuru RR, Vaysbrot E, Osani M, Turgunbaev M, Miller AS, McAlindon T (2017). 2017 American College of Rheumatology Guideline for the prevention and treatment of glucocorticoid-induced osteoporosis. Arthritis Rheumatol.

[5] Papaioannou A, Morin S, Cheung AM, Atkinson S, Brown JP, Feldman S, Hanley DA, Hodsman A, Jamal SA, Kaiser SM, Kvern B, Siminoski K, Leslie WD, Scientific Advisory Council of Osteoporosis C (2010) 2010 clinical practice guidelines for the diagnosis and management of osteoporosis in Canada: summary. CMAJ 182 (17):1864–1873. doi:10.1503/cmaj.100771, 2010 10.1503/cmaj.100771 PMC2988535 20940232

[6] Songpatanasilp T, Sritara C, Kittisomprayoonkul W, Chaiumnuay S, Nimitphong H, Charatcharoenwitthaya N, Pongchaiyakul C, Namwongphrom S, Kitumnuaypong T, Srikam W, Dajpratham P, Kuptniratsaikul V, Jaisamrarn U, Tachatraisak K, Rojanasthien S, Damrongwanich P, Wajanavisit W, Pongprapai S, Ongphiphadhanakul B, Taechakraichana N (2016). Thai Osteoporosis Foundation (TOPF) position statements on management of osteoporosis. Osteoporosis and Sarcopenia.

[7] Wu CH, McCloskey EV, Lee JK, Itabashi A, Prince R, Yu W, Li-Yu J, Chionh SB, Zhao Y, Shin CS, Gunawan T, Tsai KS, Chieng PU, Changlai SP, Chan DC, Chen JF, Tanner SB, Hans DB, Kanis JA, Chang YF, Sun ZJ, Yang RS, Asia Pacific Panel of I (2014). Consensus of official position of IOF/ISCD FRAX initiatives in Asia-Pacific region. J Clin Densitom.

[8] Royal College of Orthopaedic Surgeons of Thailand, Thai Osteoporosis Foundation (2010) Clinical practice guideline for osteoporosis. Royal College of Orthopaedic Surgeons of Thailand, Bangkok

[9] National Osteoporosis Foundation (2008) Clinician’s guide to prevention and treatment of osteoporosis, Washington DC, USA

[10] Plaza B (2011). Google Analytics for measuring website performance. Tour Manag.

[11] Google LLC Google Analytics. https://analytics.google.com/analytics/web/

[12] Official Statistic Registration Systems (2017) Population and house statistics report. Bureau of Registration Administration, Department of Provincial Administration, Ministry of Interior of Thailand,. http://stat.bora.dopa.go.th/stat/statnew/statTDD/. Accessed January 2019

[13] Worldometers Countries in the world by population (2018). http://www.worldometers.info/world-population/population-by-country/. Accessed November 29th 2018

[14] McCloskey EV, Johansson H, Harvey NC, Compston J, Kanis JA (2017). Access to fracture risk assessment by FRAX and linked National Osteoporosis Guideline Group (NOGG) guidance in the UK-an analysis of anonymous website activity. Osteoporos Int.

[15] Foundation of Thai Gerontology Research and Development Institute (TGRI) (2017). Situation of Thai elderly.

[16] Strategy and Planning Division, Office of the Permanent Secretary for Public Health, Ministry of Public Health of Thailand (2018) Detailed indicators of the Ministry of Public Health fiscal year 2018

[17] Shepstone L, Lenaghan E, Cooper C, Clarke S, Fong-Soe-Khioe R, Fordham R, Gittoes N, Harvey I, Harvey N, Heawood A, Holland R, Howe A, Kanis J, Marshall T, O’Neill T, Peters T, Redmond N, Torgerson D, Turner D, McCloskey E, Team SS (2018). Screening in the community to reduce fractures in older women (SCOOP): a randomised controlled trial. Lancet.

[18] Turner DA, Khioe RFS, Shepstone L, Lenaghan E, Cooper C, Gittoes N, Harvey NC, Holland R, Howe A, McCloskey E, O’Neill TW, Torgerson D, Fordham R, Team SS (2018). The cost-effectiveness of screening in the community to reduce osteoporotic fractures in older women in the UK: economic evaluation of the SCOOP study. J Bone Miner Res.

[19] Pongchaiyakul C, Leerapun T, Wongsiri S, Songpattanasilp T, Taechakraichana N (2012). Value and validation of RCOST and TOPF clinical practice guideline for osteoporosis treatment. J Med Assoc Thail.

[20] McCloskey E, Johansson H, Harvey NC, Shepstone L, Lenaghan E, Fordham R, Harvey I, Howe A, Cooper C, Clarke S, Gittoes N, Heawood A, Holland R, Marshall T, O’Neill TW, Peters TJ, Redmond N, Torgerson D, Kanis JA, Team SS (2018). Management of patients with high baseline hip fracture risk by FRAX reduces hip fractures-a post hoc analysis of the SCOOP study. J Bone Miner Res.

[21] Strom O, Borgstrom F, Kanis JA, Compston J, Cooper C, McCloskey EV, Jonsson B (2011). Osteoporosis: burden, health care provision and opportunities in the EU: a report prepared in collaboration with the International Osteoporosis Foundation (IOF) and the European Federation of Pharmaceutical Industry Associations (EFPIA). Arch Osteoporos.

[22] Mithal A, Ebeling P, Kyer CS (2013) The Asia-Pacific Regional Audit Epidemiology, costs & burden of osteoporosis in 2013. International Osteoporosis Foundation, Switzerland 10.4103/2230-8210.137485 PMC4138897 25143898

