# Supplementary material for: Temporal changes in access to FRAX® in Thailand between 2010 and 2018
Source: Arch Osteoporos. 2019 Jun 21;14(1):66. doi: 10.1007/s11657-019-0613-2 (PMC6588646; doi:10.1007/s11657-019-0613-2)
Supplement: Supplementary file 1 — (PDF 78 kb) [file 11657_2019_613_MOESM1_ESM.pdf]

Use of FRAX® in Thailand

Archives of Osteoporosis

Pojchong Chotiyarnwong[1,2], Nicholas C Harvey[3,4], Helena Johansson[5,6,7], Enwu Liu[7], Mattias Lorentzen[8,9], John A Kanis[5,7] and Eugene V McCloskey[2,5,10]

[1]Department of Orthopaedic Surgery, Faculty of Medicine, Siriraj Hospital, Mahidol University, Bangkok, 10700, Thailand.
[2]Academic Unit of Bone Metabolism, Department of Oncology and Metabolism, The Mellanby Centre For Bone Research, University of Sheffield, Sheffield, UK
[3]MRC Lifecourse Epidemiology Unit, University of Southampton, Southampton, UK
[4]NIHR Southampton Biomedical Research Centre, University of Southampton and University Hospital Southampton NHS Foundation Trust, Southampton, UK
[5]Centre for Metabolic Diseases, University of Sheffield Medical School, Beech Hill Road, Sheffield S10 2RX, UK
[6]Centre for Bone and Arthritis Research (CBAR), Sahlgrenska Academy, University of Gothenburg, Gothenburg, Sweden
[7]Mary MacKillop Institute for Health Research, Australian Catholic University, Melbourne, Victoria, Australia.
[8]Region Västra Götaland, Geriatric Medicine Clinic, Sahlgrenska University Hospital, Gothenburg, Sweden
[9]Geriatric Medicine, Department of Internal Medicine and Clinical Nutrition, Institute of Medicine, Sahlgrenska Academy, University of Gothenburg, Gothenburg, Sweden
[10]Centre for Integrated research into Musculoskeletal Ageing, University of Sheffield Medical School, Sheffield, UK

Correspondence

Prof. Eugene McCloskey
Email address: e.v.mccloskey@sheffield.ac.uk

Supplementary table 1. Thailand's provinces ranking of the usage of FRAX® tools

Region (Province)	Estimated 2017 population	Usage sessions	Usage sessions per 1,000 population	Ranking by FRAX® usage sessions
Bangkok	5,682,415	20,117	3.54	1
Khon Kaen	1,805,910	2,375	1.32	2
Chiang Mai	1,746,840	1,783	1.02	3
Chon Buri	1,509,125	994	0.66	4
Songkhla	1,424,230	615	0.43	5
Nonthaburi	1,229,735	445	0.36	6
Phitsanulok	865,368	389	0.45	7
Nakhon Ratchasima	2,639,226	362	0.14	8
Nakhon Pathom	911,492	328	0.36	9
Samut Prakan	1,310,766	317	0.24	10
Phuket	402,017	273	0.68	11
Chiang Rai	1,287,615	242	0.19	12
Ubon Ratchathani	1,869,633	240	0.13	13
Nakhon Si Thammarat	1,557,482	216	0.14	14
Pathum Thani	1,129,115	198	0.18	15
Chachoengsao	709,889	188	0.26	16
Nong Khai	521,886	156	0.30	17
Phra Nakhon Si Ayutthaya	813,852	150	0.18	18
Lampang	746,547	133	0.18	19
Nakhon Sawan	1,065,334	102	0.10	20
Phayao	477,100	94	0.20	21
Surat Thani	1,057,581	81	0.08	22
Nakhon Nayok	259,342	80	0.31	23
Samut Sakhon	568,465	69	0.12	24
Chanthaburi	534,459	62	0.12	25
Prachuap Khiri Khan	543,979	60	0.11	26
Phetchabun	995,331	59	0.06	27
Saraburi	642,040	56	0.09	28
Chaiyaphum	1,139,356	54	0.05	29
Trang	643,072	54	0.08	29
Krabi	469,769	51	0.11	31
Udon Thani	1,583,092	43	0.03	32
Maha Sarakham	963,072	41	0.04	33
Surin	1,397,180	37	0.03	34
Yala	527,295	32	0.06	35
Rayong	711,236	30	0.04	36
Lopburi	757,273	28	0.04	37
Kanchanaburi	887,979	24	0.03	38
Uttaradit	457,092	22	0.05	39
Mae Hong Son	279,088	21	0.08	40

Region (Province)	Estimated 2017 population	Usage sessions	Usage sessions per 1,000 population	Ranking by FRAX® usage sessions
Phrae	447,564	20	0.04	41
Ratchaburi	871,714	19	0.02	42
Suphan Buri	852,003	19	0.02	42
Tak	644,267	18	0.03	44
Si Sa Ket	210,088	18	0.09	44
Buri Ram	1,591,905	17	0.01	46
Phichit	541,868	13	0.02	47
Nong Bua Lam Phu	511,641	13	0.03	48
Narathiwat	796,239	12	0.02	49
Kalasin	986,005	11	0.01	50
Kamphaeng Phet	729,133	11	0.02	50
Nan	479,838	11	0.02	50
Roi Et	1,307,911	11	0.01	50
Sukhothai	599,319	11	0.02	50
Prachin Buri	487,544	9	0.02	55
Yasothon	539,542	7	0.01	56
Chumphon	509,650	6	0.01	57
Lamphun	405,918	6	0.01	57
Sakon Nakhon	1,149,472	6	0.01	57
Sa Kaeo	561,938	5	0.01	60
Samut Songkhram	193,902	5	0.03	60
Ang Thong	281,187	5	0.02	60
Satun	319,700	4	0.01	63
Trat	229,649	4	0.02	63
Phatthalung	524,857	3	0.01	65
Pattani	709,796	3	0.00	65
Phetchaburi	482,375	3	0.01	65
Phang-nga	267,491	3	0.01	65
Sing Buri	1,472,031	3	0.00	65
Nakhon Phanom	718,028	2	0.00	70
Amnat Charoen	378,107	2	0.01	70
Uthai Thani	329,942	2	0.01	70
Loei	641,666	1	0.00	73
Bueng Kan	423,032	1	0.00	73
Ranong	190,399	1	0.01	73
Mukdahan	350,782	1	0.00	73
Chai Nat	329,722	0	0.00	77
