# Supplementary material for: Temporal changes in access to FRAX® in Thailand between 2010 and 2018
Source: Arch Osteoporos. 2019 Jun 21;14(1):66. doi: 10.1007/s11657-019-0613-2 (PMC6588646; doi:10.1007/s11657-019-0613-2)
Supplement: Supplementary file 2 — (PDF 152 kb) [file 11657_2019_613_MOESM2_ESM.pdf]

Use of FRAX® in Thailand

Archives of Osteoporosis

Pojchong Chotiyarnwong[1,2], Nicholas C Harvey[3,4], Helena Johansson[5,6,7], Enwu Liu[7], Mattias Lorentzen[8,9], John A Kanis[5,7] and Eugene V McCloskey[2,5,10]

[1]Department of Orthopaedic Surgery, Faculty of Medicine, Siriraj Hospital, Mahidol University, Bangkok, 10700, Thailand.
[2]Academic Unit of Bone Metabolism, Department of Oncology and Metabolism, The Mellanby Centre For Bone Research, University of Sheffield, Sheffield, UK
[3]MRC Lifecourse Epidemiology Unit, University of Southampton, Southampton, UK
[4]NIHR Southampton Biomedical Research Centre, University of Southampton and University Hospital Southampton NHS Foundation Trust, Southampton, UK
[5]Centre for Metabolic Diseases, University of Sheffield Medical School, Beech Hill Road, Sheffield S10 2RX, UK
[6]Centre for Bone and Arthritis Research (CBAR), Sahlgrenska Academy, University of Gothenburg, Gothenburg, Sweden
[7]Mary MacKillop Institute for Health Research, Australian Catholic University, Melbourne, Victoria, Australia.
[8]Region Västra Götaland, Geriatric Medicine Clinic, Sahlgrenska University Hospital, Gothenburg, Sweden
[9]Geriatric Medicine, Department of Internal Medicine and Clinical Nutrition, Institute of Medicine, Sahlgrenska Academy, University of Gothenburg, Gothenburg, Sweden
[10]Centre for Integrated research into Musculoskeletal Ageing, University of Sheffield Medical School, Sheffield, UK

Correspondence

Prof. Eugene McCloskey
Email address: e.v.mccloskey@sheffield.ac.uk

Supplementary table 2. World's countries (or dependency) ranking of the usage of FRAX® tools.

Country (or dependency)	Estimated 2018 population	Land area (km^2)	Usage sessions	Usage sessions per 1,000 population	Ranking of FRAX® usage sessions
United States	326,766,748	9,147,420	4,258,344	13.03	1
United Kingdom	66,573,504	241,930	1,317,755	19.79	2
Canada	36,953,765	9,093,510	332,875	9.01	3
Spain	46,397,452	498,800	268,052	5.78	4
Slovenia	2,081,260	20,140	232,647	111.78	5
Japan	127,185,332	364,555	205,401	1.61	6
France	65,233,271	547,557	182,794	2.80	7
Sweden	9,982,709	410,340	171,250	17.15	8
Brazil	210,867,954	8,358,140	166,491	0.79	9
Belgium	11,498,519	30,280	143,426	12.47	10
Switzerland	8,544,034	39,516	140,043	16.39	11
Italy	59,290,969	294,140	121,788	2.05	12
Russia	143,964,709	16,376,870	120,731	0.84	13
Australia	24,772,247	7,682,300	116,537	4.70	14
Mexico	130,759,074	1,943,950	114,196	0.87	15
Hungary	9,688,847	90,530	90,464	9.34	16
Taiwan	23,694,089	35,410	79,971	3.38	17
Greece	11,142,161	128,900	73,651	6.61	18
Austria	8,751,820	82,409	70,364	8.04	19
Portugal	10,291,196	91,590	68,799	6.69	20
Colombia	49,464,683	1,109,500	65,177	1.32	21
China	1,415,045,928	9,388,211	63,446	0.04	22
New Zealand	4,749,598	263,310	59,622	12.55	23
Finland	5,542,517	303,890	59,538	10.74	24
Singapore	5,791,901	700	52,826	9.12	25
Poland	38,104,832	306,230	49,539	1.30	26
Ireland	4,803,748	68,890	45,054	9.38	27
South Korea	51,164,435	97,230	43,273	0.85	28
Netherlands	17,084,459	33,720	42,116	2.47	29
Argentina	44,688,864	2,736,690	41,244	0.92	30
Lebanon	6,093,509	10,230	36,195	5.94	31
Israel	8,452,841	21,640	35,381	4.19	32
Philippines	106,512,074	298,170	33,630	0.32	33
India	1,354,051,854	2,973,190	31,796	0.02	34
Thailand	69,183,173	510,890	31,617	0.46	35
Turkey	81,916,871	769,630	31,317	0.38	36
Romania	19,580,634	230,170	30,933	1.58	37
Malta	432,089	320	30,088	69.63	38
Germany	82,293,457	348,560	25,617	0.31	39
Chile	18,197,209	743,532	25,370	1.39	40
Hong Kong	7,428,887	1,050	24,267	3.27	41
Puerto Rico	3,659,007	8,870	24,237	6.62	42
Iran	82,011,735	1,628,550	19,112	0.23	43
Malaysia	32,042,458	328,550	16,368	0.51	44
Czechia	10,625,250	77,240	14,060	1.32	45
Norway	5,353,363	365,268	13,876	2.59	46
Denmark	5,754,356	42,430	11,779	2.05	47
Indonesia	266,794,980	1,811,570	11,486	0.04	48
Saudi Arabia	33,554,343	2,149,690	10,552	0.31	49
United Arab Emirates	9,541,615	83,600	10,424	1.09	50
Peru	32,551,815	1,280,000	10,100	0.31	51
Venezuela	32,381,221	882,050	8,225	0.25	52
Slovakia	5,449,816	48,088	8,105	1.49	53
South Africa	57,398,421	1,213,090	7,639	0.13	54

Country (or dependency)	Estimated 2018 population	Land area (km^2)	Usage sessions	Usage sessions per 1,000 population	Ranking of FRAX® usage sessions
Ukraine	44,009,214	579,320	6,657	0.15	55
Ecuador	16,863,425	248,360	5,823	0.35	56
Bermuda	61,070	50	5,548	90.85	57
Estonia	1,306,788	42,390	5,528	4.23	58
Bolivia	11,215,674	1,083,300	5,506	0.49	59
Vietnam	96,491,146	310,070	4,679	0.05	60
Costa Rica	4,953,199	51,060	4,403	0.89	61
Cyprus	1,189,085	9,240	3,642	3.06	62
Morocco	36,191,805	446,300	3,260	0.09	63
Croatia	4,164,783	55,960	3,176	0.76	64
Serbia	8,762,027	87,460	3,171	0.36	65
Egypt	99,375,741	995,450	3,100	0.03	66
Sri Lanka	20,950,041	62,710	3,059	0.15	67
Kuwait	4,197,128	17,820	2,989	0.71	68
Pakistan	200,813,818	770,880	2,824	0.01	69
Bangladesh	166,368,149	130,170	2,684	0.02	70
Guatemala	17,245,346	107,160	2,496	0.14	71
Georgia	3,907,131	69,490	2,470	0.63	72
Jordan	9,903,802	88,780	2,164	0.22	73
Uruguay	3,469,551	175,020	2,061	0.59	74
Belarus	9,452,113	202,910	2,011	0.21	75
Bulgaria	7,036,848	108,560	1,782	0.25	76
Panama	4,162,618	74,340	1,658	0.40	77
Luxembourg	590,321	2,590	1,632	2.76	78
Qatar	2,694,849	11,610	1,631	0.61	79
Lithuania	2,876,475	62,674	1,589	0.55	80
Kazakhstan	18,403,860	2,699,700	1,553	0.08	81
Guernsey	66,502	65	1,525	22.93	82
Iceland	337,780	100,250	1,470	4.35	83
Tunisia	11,659,174	155,360	1,082	0.09	84
Bosnia & Herzegovina	3,503,554	51,000	1,042	0.30	85
El Salvador	6,411,558	20,720	928	0.14	86
Algeria	42,008,054	2,381,740	819	0.02	87
Oman	4,829,946	309,500	797	0.17	88
Jersey	166,083	120	770	4.64	89
Moldova	4,041,065	32,850	766	0.19	90
Myanmar (Burma)	53,855,735	653,290	749	0.01	91
Armenia	2,934,152	28,470	686	0.23	92
Iraq	39,339,753	434,320	672	0.02	93
Palestine	5,052,776	6,020	656	0.13	94
Dominican Republic	10,882,996	48,320	601	0.06	95
Macau	632,418	30	593	0.94	96
Latvia	1,929,938	62,200	572	0.30	97
Réunion	883,247	2,500	561	0.64	98
Syria	18,284,407	183,630	560	0.03	99
Guam	165,718	540	541	3.26	100
Paraguay	6,896,908	397,300	505	0.07	101
Trinidad & Tobago	1,372,598	5,130	505	0.37	102
Nepal	29,624,035	143,350	501	0.02	103
Kenya	50,950,879	569,140	488	0.01	104
Honduras	9,417,167	111,890	483	0.05	105
Albania	2,934,363	27,400	438	0.15	106
Jamaica	2,898,677	10,830	404	0.14	107
Nicaragua	6,284,757	120,340	361	0.06	108
Bahrain	1,566,993	760	345	0.22	109
Isle of Man	84,831	570	334	3.94	110

Country (or dependency)	Estimated 2018 population	Land area (km^2)	Usage sessions	Usage sessions per 1,000 population	Ranking of FRAX® usage sessions
French Guiana	289,763	82,200	311	1.07	111
Macedonia (FYROM)	2,085,051	25,220	264	0.13	112
Cambodia	16,245,729	176,520	218	0.01	113
Cayman Islands	62,348	240	208	3.34	114
Kyrgyzstan	6,132,932	191,800	204	0.03	115
Nigeria	195,875,237	910,770	202	0.00	116
Mongolia	3,121,772	1,553,560	182	0.06	117
Martinique	385,065	1,060	176	0.46	118
Azerbaijan	9,923,914	82,658	155	0.02	119
Libya	6,470,956	1,759,540	148	0.02	120
Uzbekistan	32,364,996	425,400	143	0.00	121
Mauritius	1,268,315	2,030	135	0.11	122
Monaco	38,897	1	129	3.32	123
Bahamas	399,285	10,010	127	0.32	124
Sudan	41,511,526	1,765,048	118	0.00	125
Montenegro	629,219	13,450	118	0.19	125
Guadeloupe	449,173	1,690	117	0.26	127
Brunei	434,076	5,270	111	0.26	128
Senegal	16,294,270	192,530	110	0.01	129
Liechtenstein	38,155	160	105	2.75	130
Gibraltar	34,733	10	85	2.45	131
Barbados	286,388	430	85	0.30	131
Cuba	11,489,082	106,440	81	0.01	133
Andorra	76,953	470	79	1.03	134
Ethiopia	107,534,882	1,000,000	75	0.00	135
Zimbabwe	16,913,261	386,850	74	0.00	136
Uganda	44,270,563	199,810	74	0.00	136
San Marino	33,557	60	70	2.09	138
New Caledonia	279,821	18,280	67	0.24	139
Cameroon	24,678,234	472,710	65	0.00	140
Yemen	28,915,284	527,970	64	0.00	141
U.S. Virgin Islands	104,914	350	60	0.57	142
Ghana	29,463,643	227,540	57	0.00	143
French Polynesia	285,859	3,660	56	0.20	144
Laos	6,961,210	230,800	55	0.01	145
Namibia	2,587,801	823,290	47	0.02	146
Curaçao	161,577	444	40	0.25	147
St. Helena	4,074	390	40	9.82	147
Tanzania	59,091,392	885,800	39	0.00	149
Botswana	2,333,201	566,730	39	0.02	150
Aruba	105,670	180	35	0.33	151
Fiji	912,241	18,270	35	0.04	151
Kosovo	118,414	810	30	0.25	153
Maldives	444,259	300	30	0.07	153
Côte d'Ivoire	24,905,843	318,000	28	0.00	155
Suriname	568,301	156,000	27	0.05	156
Guyana	782,225	196,850	25	0.03	157
Grenada	108,339	340	24	0.22	158
Belize	382,444	22,810	24	0.06	158
Falkland Islands (Islas Malvinas)	49,489	1,396	24	0.48	158
Turks & Caicos Islands	35,963	950	23	0.64	161
Mozambique	30,528,673	786,380	22	0.00	162
Zambia	17,609,178	743,390	22	0.00	162
Tajikistan	9,107,211	139,960	19	0.00	164
Cape Verde	553,335	4,030	19	0.03	164

Country (or dependency)	Estimated 2018 population	Land area (km²)	Usage sessions	Usage sessions per 1,000 population	Ranking of FRAX® usage sessions
Angola	30,774,205	1,246,700	18	0.00	166
Afghanistan	36,373,176	652,860	18	0.00	166
Antigua & Barbuda	103,050	440	17	0.16	168
Haiti	11,112,945	27,560	16	0.00	169
Guinea	13,052,608	245,720	16	0.00	169
St. Vincent & Grenadines	110,200	390	15	0.14	171
Madagascar	26,262,810	581,795	14	0.00	172
Congo - Kinshasa	84,004,989	2,267,050	14	0.00	172
Sint Maarten	40,552	34	13	0.32	174
Dominica	74,308	750	13	0.17	174
Anguilla	15,045	90	13	0.86	174
Northern Mariana Islands	55,194	460	13	0.24	174
Turkmenistan	5,851,466	469,930	13	0.00	174
Malawi	19,164,728	94,280	12	0.00	179
Mali	19,107,706	1,220,190	11	0.00	180
Caribbean Netherlands	25,702	328	11	0.43	180
Rwanda	12,501,156	24,670	10	0.00	182
St. Kitts & Nevis	55,850	260	10	0.18	182
St. Lucia	179,667	610	9	0.05	184
Faroe Islands	2,922	12,170	8	2.74	185
Micronesia	106,227	700	8	0.08	186
Benin	11,485,674	112,760	7	0.00	187
Bhutan	817,054	38,117	7	0.01	187
Greenland	56,565	410,450	6	0.11	189
Togo	7,990,926	54,390	6	0.00	189
Vatican City	801	0.44	6	7.49	189
British Virgin Islands	31,719	150	6	0.19	189
Mauritania	4,540,068	1,030,700	6	0.00	189
Somalia	15,181,925	627,340	6	0.00	189
Burkina Faso	19,751,651	273,600	5	0.00	195
Djibouti	971,408	23,180	5	0.01	195
Congo - Brazzaville	5,399,895	341,500	5	0.00	195
Papua New Guinea	8,418,346	452,860	4	0.00	198
Swaziland	1,391,385	17,200	4	0.00	198
Gambia	2,163,765	10,120	4	0.00	198
Gabon	2,067,561	257,670	4	0.00	198
Timor-Leste	1,324,094	14,870	3	0.00	202
St. Pierre & Miquelon	6,342	230	3	0.47	202
South Sudan	12,919,053	610,952	3	0.00	202
Mayotte	259,682	375	3	0.01	202
Palau	21,964	460	3	0.14	202
Comoros	832,347	1,861	3	0.00	202
Tonga	109,008	720	3	0.03	202
Lesotho	2,263,010	30,360	2	0.00	209
Liberia	4,853,516	96,320	2	0.00	209
Vanuatu	282,117	12,190	2	0.01	209
Seychelles	95,235	460	2	0.02	209
Samoa	197,695	2,830	2	0.01	209
Norfolk Island	1,748	34.6	2	1.14	209
St. Martin	40,120	87	2	0.05	209
Svalbard & Jan Mayen	no permanent population	61,400	2	n/a	209
Niger	22,311,375	1,266,700	1	0.00	217
Burundi	11,216,450	25,680	1	0.00	217
Marshall Islands	53,167	180	1	0.02	217
Chad	15,353,184	1,259,200	1	0.00	217

Country (or dependency)	Estimated 2018 population	Land area (km^2)	Usage sessions	Usage sessions per 1,000 population	Ranking of FRAX® usage sessions
Central African Republic	4,737,423	622,980	1	0.00	217
Eritrea	5,187,948	101,000	1	0.00	217
Equatorial Guinea	1,313,894	28,050	1	0.00	217
Montserrat	5,203	100	1	0.19	217
Solomon Islands	623,281	27,990	1	0.00	217
Guinea-Bissau	1,907,268	28,120	0	0.00	226
American Samoa	55,679	200	0	0.00	226
St. Barthélemy	9,427	25	0	0.00	226
Channel Islands	166,083	190	n/a	n/a	n/a
Cook Islands	17,411	240	n/a	n/a	n/a
Nauru	11,312	20	n/a	n/a	n/a
Niue	1,624	260	n/a	n/a	n/a
North Korea	25,610,672	120,410	n/a	n/a	n/a
Sao Tome & Principe	208,818	960	n/a	n/a	n/a
Sierra Leone	7,719,729	72,180	n/a	n/a	n/a
Tokelau	1,319	10	n/a	n/a	n/a
Tuvalu	11,287	30	n/a	n/a	n/a
Wallis & Futuna	11,683	140	n/a	n/a	n/a
Western Sahara	567,421	266,000	n/a	n/a	n/a
